# Towards eliminating malaria in high endemic countries: the roles of community health workers and related cadres and their challenges in integrated community case management for malaria: a systematic review

**DOI:** 10.1186/s12936-016-1667-x

**Published:** 2017-01-03

**Authors:** Bruno F. Sunguya, Linda B. Mlunde, Rakesh Ayer, Masamine Jimba

**Affiliations:** 1 Department of Community Health, School of Public Health and Social Sciences, Muhimbili University of Health and Allied Sciences, Dar es Salaam, Tanzania; 2 Management for Development and Health, Dar es Salaam, Tanzania; 3 Department of Community and Global Health, The University of Tokyo, Tokyo, Japan

**Keywords:** Community health workers, Malaria, Community case management, Malaria endemicity

## Abstract

**Background:**

Human resource for health crisis has impaired global efforts against malaria in highly endemic countries. To address this, the World Health Organization (WHO) recommended scaling-up of community health workers (CHWs) and related cadres owing to their documented success in malaria and other disease prevention and management. Evidence is inconsistent on the roles and challenges they encounter in malaria interventions. This systematic review aims to summarize evidence on roles and challenges of CHWs and related cadres in integrated community case management for malaria (iCCM).

**Methods:**

This systematic review retrieved evidence from PubMed, CINAHL, ISI Web of Knowledge, and WHO regional databases. Terms extracted from the Boolean phrase used for PubMed were also used in other databases. The review included studies with Randomized Control Trial, Quasi-experimental, Pre-post interventional, Longitudinal and cohort, Cross-sectional, Case study, and Secondary data analysis. Because of heterogeneity, only narrative synthesis was conducted for this review.

**Results:**

A total of 66 articles were eligible for analysis out of 1380 studies retrieved. CHWs and related cadre roles in malaria interventions included: malaria case management, prevention including health surveillance and health promotion specific to malaria. Despite their documented success, CHWs and related cadres succumb to health system challenges. These are poor and unsustainable finance for iCCM, workforce related challenges, lack of and unsustainable supply of medicines and diagnostics, lack of information and research, service delivery and leadership challenges.

**Conclusions:**

Community health workers and related cadres had important preventive, case management and promotive roles in malaria interventions. To enable their effective integration into the health systems, the identified challenges should be addressed. They include: introducing sustainable financing on iCCM programmes, tailoring their training to address the identified gaps, improving sustainable supply chain management of malaria drugs and diagnostics, and addressing regulatory challenges in the local contexts.

## Background

Mortality among children under 5 years old has fallen by more than 50% in the last decade [1]. However, the global burden of diseases and years of life lost are still high in low and middle-income countries owing to infectious diseases, including malaria [1]. Malaria burden remains high despite the knowledge of effective interventions [2]. Such interventions include community-based approaches for prevention and treatment of common illnesses responsible for high mortality and morbidity, such as malaria [3–5].

Community-based interventions call for individuals available in and originated from the respective communities to implement them. Community health workers (CHWs) have been effective in improving access to preventative, promotive and curative interventions in the communities they serve [6]. In malaria interventions, CHWs and related cadres have improved outcomes in disease control by tailoring interventions to local needs and regulations. The World Health Organization (WHO) has endorsed CHW-led interventions and encouraged its member states to embrace integrated community case management (iCCM) approaches and policies to address child mortality [7].

The iCCM approach using CHWs and related cadres has been effective in managing and preventing child deaths due to malaria in various contexts [6, 8]. Their use is cost-effective [9]. However, more than half a million children still die of malaria every year [1]. Drug resistance and mutation of the malaria parasite have presented significant hurdles in decreasing the persistently high mortality rates of malaria in children, particularly in highly endemic regions. Such complex factors in disease transmission and treatment present particularly difficult challenges for the iCCM approach, which relies on less-trained CHWs and related cadres who may have elementary skills and knowledge in malaria. They may not be able to manage more complex cases present to them.

Implementation of iCCM interventions has encountered various challenges. They have included shortages of drugs and supplies, poor quality of care, and lack of CHW incentives, training and supervision [8]. Such challenges continue to risk stalling positive outcomes obtained through iCCM interventions. In particular, they risk the establishment, scale-up and sustainability of iCCM interventions in reducing child mortality. In some settings, CHWs in iCCM programmes have been tasked with roles beyond what they are trained to do [7, 10]. Lack of health workers has influenced task-shifting from qualified medical personnel to CHWs for malaria case management as the only alternative. In other areas, where CHWs are the only personnel available, they have been used to deliver effective life-saving interventions [4].

Success of iCCM using CHWs and related cadres has been well documented. However, evidence of challenges and differing roles of CHWs and other lay health workers in various endemic regions has not been systematically examined. Challenges learnt from such varied implementation locations may help the process of adaptation of iCCM interventions in areas with similar characteristics. This systematic review was conducted to examine and summarize evidence on different roles of CHWs and related cadres in malaria prevention, case management and health promotion in malaria-endemic regions. This review also aimed to examine the challenges encountered by such health cadres in the implementation of iCCM.

## Methods

This systematic review aimed to address two Population Intervention Comparator Outcome (PICO) questions: What is the role of CHWs and related cadres in malaria prevention, case management and health promotion in highly malaria-endemic regions? and, What are the challenges encountered while implementing iCCM for malaria using CHWs and related cadres?

In this review, the population of interest included CHWs and related cadres, such as village health volunteers and other lay health workers: home care providers and community medicine distributors. Qualified health cadres or those who had more formal and qualified training were excluded from this study. This also included mid-level providers and other official health workers employed to provide care in health facilities. Interventions of interest included iCCM, community case management of malaria (CCMm), seasonal malaria chemoprevention (SMC), and home-based management of fever. This review did not include a comparison group because of the nature of the two PICO questions.

The outcome of interest for this review was the roles and challenges faced by CHWs and related cadres. Challenges of CHWs and the related cadres were defined in line with the health system building blocks put forth by WHO [11]. They were grouped into financing, workforce, medical products, information and research, service delivery, and stewardship.

The developed protocol was registered in the PROSPERO database for systematic reviews (Registration number CRD42015027878). The current review is set to answer two of the four research objectives in the registered protocol. These are examining roles and challenges encountered by CHWs working in malaria interventions in malaria-endemic regions. Evidence search was conducted in PubMed, CINAHL, ISI Web of Knowledge, and WHO regional databases. A Boolean phrase was prepared and used for evidence search in PubMed, while search terms were used in other databases. Studies with the following designs were included: randomized control trial; quasi-experimental; pre-post interventional; longitudinal and cohort; cross-sectional; case study; and, secondary data analysis. Evidence in form of opinion papers, reviews, editorials, and reports was excluded in this review.

**Fig. 1:**
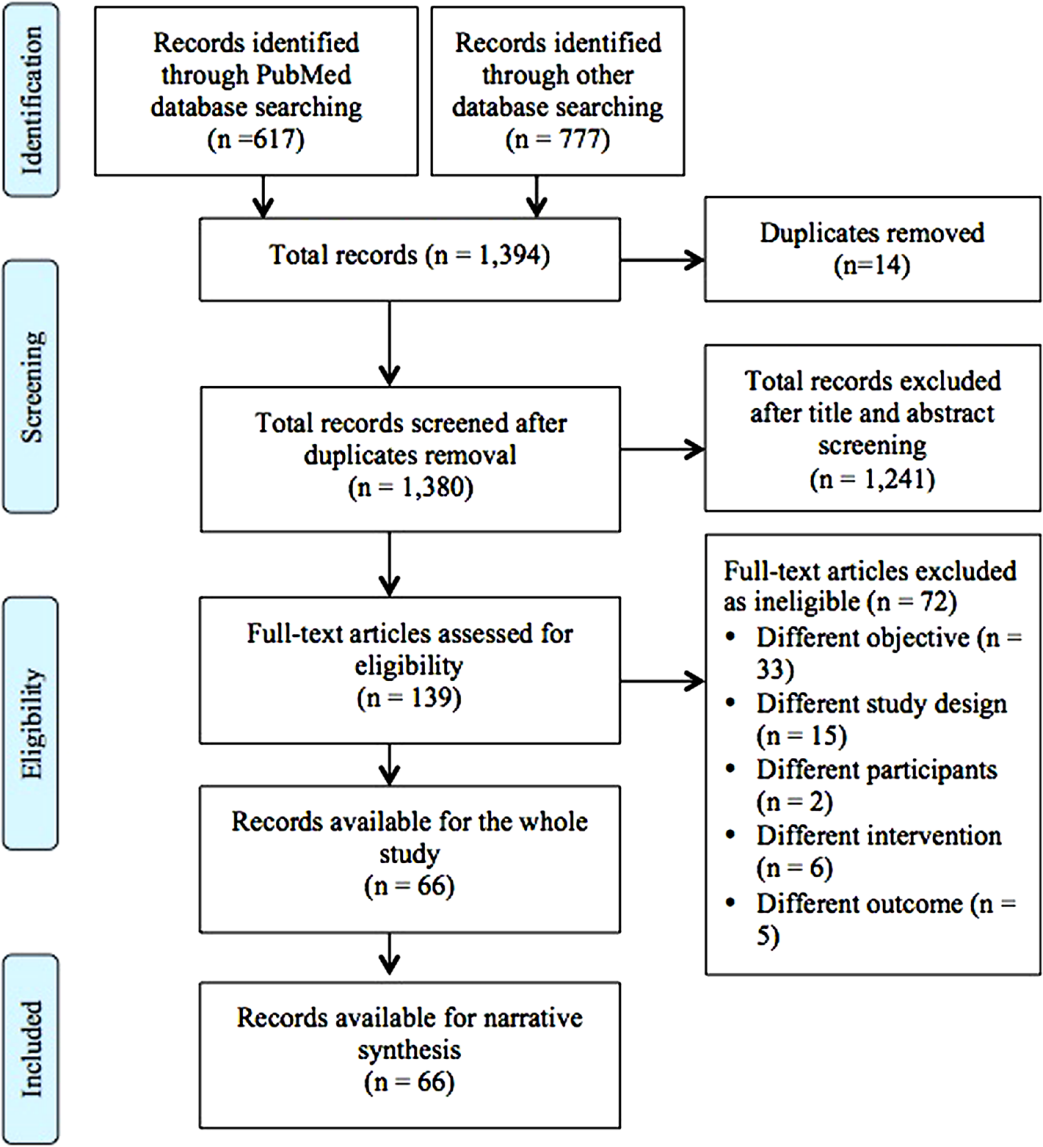
PRISMA flow chart through phases of systematic review

A total of 1394 articles were retrieved. Of them, 617 articles were identified from PubMed and 777 articles from all other databases (Fig. 1). A total of 1380 were screened after removal of 14 articles as duplicates. Of the remaining, 1245 articles were further excluded based on their titles and abstracts. Only 139 articles were eligible for full text assessment based on inclusion and exclusion criteria. On the full text assessment, a total of 72 articles were further excluded based on differences in objectives (n = 33), study design (n = 15), participants (n = 2), interventions (n = 6), outcomes (n = 5), and lack of the defined intervention (n = 11). Finally, a total of 68 articles were eligible for analysis. Excel spreadsheet was used to report the extracted data. Only a narrative synthesis on the included studies was conducted because of the differences in study designs and measurements of outcome variables.

## Results

### Description of the selected studies

This review retrieved studies conducted in regions with high malaria endemicity (Table 1). These included Southeast Asia and sub-Saharan Africa regions. In the retrieved studies, CHWs were the commonest health cadre in 38 studies. Others included community health volunteers, village malaria workers, community medicine distributors, village health workers, home care providers, accredited social health activists, volunteer community-directed distributors, health surveillance assistants, village volunteers, community-owned resource persons, drug shop attendants, drug shop vendors, traditional birth attendants, community reproductive health workers, adolescent peer mobilizers, volunteer health workers, volunteer collaborators, women leaders, and mothers. In sub-Saharan Africa, the commonest cadre was CHW, while in Asia it was village malaria worker.

**Table 1:** Description of the studies included in the review

No	Citation	Country	Study design	Intervention	Cadre
1.	Rodriguez et al. [20]	Malawi	Case study	iCCM	Health surveillance assistants
2.	Chilundo et al. [21]	Mozambique	Qualitative study	iCCM	CHWs
3.	Yansaneh et al. [33]	Sierra Leone	Mixed methods: household survey, in-depth interviews, focus group discussions	Free healthcare initiative and iCCM	CHVs
4.	Witek-McManus et al. [34]	Malawi	Pre-post interventional study	Training programme for school teachers	CHWs
5.	Nanyonjo et al. [30]	Uganda	Cross-sectional study	iCCM	CHWs
6.	Heidkamp et al. [26]	Malawi	Cross-sectional study	iCCM	CHWs, called health surveillance assistants
7.	Linn et al. [19]	Senegal	Quasi-experimental study	ProAct model (iCCM in which CHWs proactively search for cases)	HCPs
8.	Druetz et al. [35]	Burkina Faso	Cross-sectional study	Community case management of malaria	CHWs
9.	Das et al. [36]	India	Pre-post interventional study	a. Supportive supervision of ASHA plus community mobilization b. Community mobilization only	ASHA
10.	Yansaneh et al. [12]	Sierra Leone	Pre-post interventional study	Health for the poorest quintile, focussing on 3 diseases: diarrhoea, malaria, pneumonia.	CHWs
11.	Banek et al. [13]	Uganda	Mixed methods: cross-sectional, qualitative design	Home-base management of fever	CMDs
12.	Hamainza et al. [22]	Zambia	Longitudinal study	CHWs providing passive and active visits to households	CHWs
13.	Abbey et al. [24]	Ghana	Mixed method: cross-sectional, qualitative design	Community-based health intervention	CHWs
14.	Lwin et al. [37]	Myanmar	Community-base intervention study	Sun primary health community-based intervention	CHWs
15.	Tine et al. [14]	Senegal	Randomized controlled trial	CCMm and seasonal malaria chemoprevention	CHWs
16.	Tine et al. [29]	Senegal	Randomized controlled trial	Home-based management of malaria using RDT, ACT, rectal artesunate seasonal malaria chemoprevention delivered by CHWs	CHWs
17.	Nanyonjo et al. [18]	Uganda	Cross-sectional study	iCCM	CHWs: *Primary health facility workers (PFHWs)*
18.	Siekmans et al. [38]	Kenya	Cross-sectional study	iCCM	CHWs
19.	Ndiaye et al. [39]	Senegal	Secondary data analysis	CCMm	CHWs
20.	Blanas et al. [28]	Senegal	Mixed-methods design	CCMm	CHWs
21.	Ohnmar et al. [40]	Myanmar	Randomized controlled trial	Training unpaid village volunteers in provision of RDT, ACT and supervision	Village volunteers
22.	Lim et al. [41]	Cambodia	Cross-sectional study	VMW *vs* health facility health worker intervention	VMW
23.	Kisia et al. [42]	Kenya	Cross-sectional study	CCMm	CHWs
24.	Counihan et al. [25]	Zambia	Longitudinal study	CHW intervention	CHWs
25.	Rutta et al. [43]	Tanzania	Pre-post intervention study	CORPs to provide early diagnosis and treatment of malaria	CORPs
26.	Ratsimbasoa et al. [44]	Madagascar	Mixed methods design	RDTs conducted by CHWs, compared to PCR and microscopy	CHWs
27.	Brenner et al. [23]	Uganda	Pre-post intervention study	Volunteer community health worker intervention	Community health volunteers
28.	Mukanga et al. [45]	Uganda	Qualitative study	Integrated malaria and pneumonia community case management	CHWs
29.	Thiam et al. [46]	Senegal	Secondary data analysis	Home-based management of malaria	HCPs
30.	Okeibunor et al. [15]	Nigeria	Pre-post intervention study	VCDDs intervention	VCDD
31.	Lemma et al. [47]	Ethiopia	Pre-post intervention study	Training of CHWs	CHWs
32.	Patouillard et al. [16]	Ghana	Randomized controlled trial	Intermittent preventive treatment of malaria in children (IPTc)	Community health volunteers
33.	Chanda et al. [48]	Zambia	Cross-sectional study	HMM	CHWs
34.	Chanda et al. [49]	Zambia	Prospective study	CHWs intervention	CHWs
35	Ngasala et al. [50]	Tanzania	Prospective study	Delivery of artemether–lumefantrine by community health workers	CHWs
36.	Phommanivong et al. [51]	Lao PDR	Prospective study	Training of village health volunteers	Village health workers
37.	Yeboah-Antwi et al. [52]	Zambia	Cluster randomized controlled trial	CHW intervention	CHWs
38.	Mukanga et al. [53]	Uganda	Qualitative study	CHW intervention	CMDs
39.	Yasuoka et al. [17]	Cambodia	Cross-sectional study	VMW intervention	VMW
40.	Hawkes et al. [54]	Democratic Republic of Congo	Prospective cohort study	Training of CHWs	CHWs
41.	Eke et al. [55]	Nigeria	Prospective cohort study	CHW intervention	CHWs
42.	Awor et al. [56]	Uganda	Quasi-experimental study	iCCM	Drug shop attendants
43.	Cox et al. [57]	Cambodia	Mixed methods study	Community-based surveillance systems	VMW
44.	Hamainza et al. [22]	Zambia	Cross-sectional study	Mobile phone SMS vs register book	CHWs
45.	Ndiaye et al. [58]	Senegal	Prospective cohort study	Paediatric kit containing quinine, purified water, syringe	CHWs
46.	Das et al. [59]	India	Longitudinal study	Community-based presumptive chloroquine treatment	Volunteers
47.	Mbonye et al. [60]	Uganda	Intervention study	Community-based IPTp	Drug shop vendors, traditional birth attendants, community reproductive health worker, adolescent peer mobilizer
48.	Vanek et al. [61]	Tanzania	Cross-sectional study	Community-based surveillance	CORPs
49.	Cho-Min-Naing et al. [62]	Myanmar	Cross-sectional study	Rapid on-site immunochromatographic test	Volunteer health workers
50.	Kelly et al. [63]	Kenya	Cross-sectional study	Community initiatives for child survival	CHWs
51.	Ruebush et al. [64]	Guatemala	Intervention study	Community-based malaria case detection system—Volunteer collaboration network (VCN)	Volunteer collaborators
52.	Aung et al. [65]	Myanmar	Pre-post intervention study	Training of CHWs	CHWs
53.	Gidebo et al. [66]	Ethiopia	Mixed-methods study	Health extension programme	CHWs
54.	Kalyango et al. [67]	Uganda	Mixed methods study	iCCM of childhood illnesses	CHWs
55.	Hamer et al. [68]	Zambia	Cluster randomized controlled trial	Training of CHWs	CHWs
56.	Mubi et al. [10]	Tanzania	Randomized cross-over trial	Training of CHWs	CHWs
57.	Harvey et al. [69]	Zambia	Quasi-experimental study	Training of CHWs	CHWs
58.	Delacollette et al. [70]	Zaire	Prospective cohort study	Training of CHWs	CHWs
59.	Eriksen et al. [71]	Tanzania	Randomized controlled trial	Training of community women leaders	Women leaders
60.	Kouyaté et al. [72]	Burkina Faso	Randomized controlled trial	Training of women group leaders by health workers	Lay community women leaders
61.	Onwujekwe et al. [73]	Nigeria	Prospective study	Training of CHWs	CHWs
62.	Mayxay et al. [74]	Laos PDR	Longitudinal study	Training of VHVs	VHVs
63.	Hii et al. [75]	Malaysia	Cross-sectional study	Community participation health programme (*Sukarelawan Penjagaan Kesihatan Primer* (SPKP))	VHVs
64.	Spencer et al. [76]	Kenya	Cross-sectional study	Community-based malaria control programme	Volunteer community health workers
65.	Ajayi et al. [77]	Nigeria	Pre-post intervention study	Training of mother trainers	CHWs
66.	Kweku et al. [78]	Ghana	Randomized controlled trial	IPTc	Community volunteers vs health workers in health facilities

*iCCM* integrated community case management, *CHWs* community health workers, *ASHA* accredited social health activist, *HCPs* home care providers, *CMDs* community medicine distributors, *VMWs* village malaria workers, *CORPs* community-owned resource persons, *CCDD* volunteer community-directed distributor, *VHVs* village health volunteers, *CHVs* community health volunteers

### Role of CHWs and related cadres in malaria interventions

Table 2 shows the different roles of CHWs and related cadres on malaria interventions. This review classified their roles into three main categories: malaria case management, prevention including health surveillance and health promotion specific to malaria. Such roles were reported in a total of 40 articles.

**Table 2:** Roles of CHWs, VMWs and lay personnel working on malaria

SN	Citation	Cadre	Roles
1.	Rodriguez et al. [20]	Health surveillance assistants	Treatment with ACT Disease surveillance Health promotion
2.	Chilundo et al. [21]	CHWs: *Agentes Polivalentes Elementares (APEs)*	Prescription of anti-malarial Management of malaria cases
3.	Yansaneh et al. [33]	Community health volunteers	Malaria treatment Health promotion Referral of critical patients or those with danger signs Accompanies malaria-sick patients to health facilities
4.	Witek-McManus et al. [34]	CHWs	Diagnosis using RDT Treatment using ACT
5.	Nanyonjo et al. [30]	CHWs	Diagnosis Patients’ referral
6.	Linn et al. [19]	HCPs	Home visitation and health promotion
7.	Druetz et al. [35]	CHWs	Patients consultations Prescription and treatment
8.	Das et al. [36]	ASHA	Patients consultations Prescription and treatment
9.	Yansaneh et al. [12]	Community health volunteers	Malaria treatment Disease prevention
10.	Banek et al. [13]	(CMDs)	Home-based treatment of malaria
11.	Hamainza et al. [22]	CHWs	Malaria treatment Diagnosis using RDT
12.	Abbey et al. [24]	CHWs	Health promotion
13.	Tine et al. [14]	CHWs	Malaria treatment Health promotion
14.	Tine et al. [29]	CHWs	Home-based treatment and diagnosis
15.	Nanyonjo et al. [18]	Primary health facility workers (PFHWs)	Facility treatment Health promotion and prevention
16.	Siekmans et al. [38]	CHWs	Home-based treatment and diagnosis
17.	Ndiaye et al. [39]	CHWs	Consultations Treatment using ACT Patients’ referrals Diagnosis using RDT
18.	Blanas et al. [28]	CHWs	Treatment and prescription of ACT Diagnosis with RDT Selling anti-malarials at government prices
19.	Ohnmar et al. [40]	Village volunteers	Treatment and prescription of ACT Diagnosis with RDT
20.	Lim et al. [41]	Village malaria workers	Diagnosis
21.	Kisia et al. [42]	CHWs	Treatment and prescription of ACT
22.	Counihan et al. [25]	CHWs	Diagnosis using RDT
23.	Rutta et al. [43]	CORPs	Diagnosis using RDT Treatment using ACT Referral of malaria cases
24.	Ratsimbasoa et al. [44]	CHWs	Diagnosis using RDT
25.	Brenner et al. [23]	Community health volunteers	Diagnosis using RDT Treatment using ACT
26.	Mukanga et al. [45]	CHWs	Patients’ consultation: taking history Diagnosis with RDT Patient’s classification
27.	Thiam et al. [46]	HCPs	Patients’ consultation: taking history Diagnosis with RDT Treatment
28.	Okeibunor et al. [15]	CDDs	Distribution of ITNs Provision of IPTp drugs Counselling services on prevention among pregnant women
29.	Lemma et al. [47]	CHWs	Diagnosis using RDT Treatment of malaria
30.	Patouillard et al. [16]	Community health volunteers	Intermittent preventive treatment in children (IPTc)
31.	Chanda et al. [48]	CHWs	Diagnosis
32.	Chanda et al. [49]	CHWs	Treatment using anti-malarials
33.	Ngasala et al. [50]	CHWs	Treatment using anti-malarials (ACT)
34.	Phommanivong et al. [51]	Village health workers	Diagnosis using RDT Treatment of malaria
35.	Yeboah-Antwi et al. [52]	CHWs	Diagnosis using RDT Treatment using ACT
36.	Mukanga et al. [53]	CMDs	Diagnosis using RDT
37.	Yasuoka et al. [17]	Village malaria workers	Diagnosis with RDTs Prescribing anti-malarials Active detection Explanations about compliance Follow-up of patients
38.	Hawkes et al. [54]	CHWs	Diagnosis using RDT Treatment of febrile conditions/malaria
39.	Eke et al. [55]	CHWs	Diagnosis using RDT
40.	Tipke et al.	Volunteer community health workers	Treatment using modern medicine
41.	Awor et al. [56]	Drug shop attendants	Malaria testing with RTD Malaria treatment with ACT
42.	Cox et al. [57]	Village malaria workers	Surveillance of day 3-positive *Plasmodium falciparum* cases
43.	Hamainza et al. [22]	CHWs	Diagnosis using RDT
44.	Ndiaye et al. [58]	CHWs	Use of paediatric kit containing quinine, purified water, syringe
45.	Das et al. [59]	Volunteers	Cases of fever treated during the 3-year period
46.	Mbonye et al. [60]	Drug shop vendors, traditional birth attendants, community reproductive health worker, adolescent peer mobilizer	Delivery of SP doses to pregnant women
47.	Vanek et al. [61]	CORPs	Number of malaria vector larval habitats
48.	Cho-Min-Naing et al. [62]	Volunteer health workers	Sensitivities of malaria parasites tests
49.	Kelly et al. [63]	CHWs	Treatment
50.	Ruebush et al. [64]	Volunteer collaborators	Number of patients treated
51.	Aung et al. [65]	CHWs	Diagnosis and treatment of paediatric malaria
52.	Gidebo et al. [66]	CHWs	Diagnosis and treatment
53.	Kalyango et al. [67]	CHWs	Treatment
54.	Hamer et al. [68]	CHWs	Use of RDT
55.	Mubi et al. [10]	CHWs	Provision of ACT
56.	Harvey et al. [69]	CHWs	Use of RDT
57.	Delacollette et al. [70]	CHWs	Treatment
58.	Phommanivong et al. [51]	Village health volunteers	Use of RDT Provision of ACT
59.	Eriksen et al. [71]	Women leaders	Role of women leaders in recognizing symptoms and providing first-line treatment for uncomplicated malaria
60.	Kouyaté et al. [72]	Lay community women leaders	Malaria case management
61.	Onwujekwe et al. [73]	CHWs	Malaria treatment
62.	Mayxay et al. [74]	Village health volunteers	Use of RDT
63.	Hii et al. [75]	Village health volunteers	Treatment
64.	Spencer et al. [76]	Volunteer community health workers	Treatment with chloroquine
65.	Ajayi et al. [77]	CHWs	Health promotion Treatment of malaria
66.	Kweku et al. [78]	Community volunteers *vs* health workers in health facilities	Administration of amodiaquine plus SP

*CHWs* community health workers, *ASHA* accredited social health activist, *HCPs* home care providers, *CMDs* community medicine distributors, *VMWs* village malaria workers, *CORPs* community-owned resource persons, *CCDD* volunteer community-directed distributor, *VHVs* village health volunteers

In malaria case management, CHWs and related cadres were involved in the diagnosis of malaria using rapid diagnostic tests (RDT). They were also involved in management of fever and the treatment of malaria using artemisinin combination therapy (ACT). In some studies, CHWs and related cadres were involved in prescription of anti-malarial drugs, delivery of anti-malarial drugs for home-based care and treatment or referral of complicated cases to the health facilities. In some cases they were the vital person in the community to accompany community members to seek care [12], or to provide home-based visitations for follow-up [13, 14] (Table 2).

Community health workers and related cadres were also involved in malaria preventive roles as shown in a few selected studies. Such roles included provision of intermittent preventive treatment for pregnant women (IPTp) [15] and for children (IPTc) [16]. CHWs and related cadres were also involved in distribution of insecticide-treated bed nets as one of the malaria prevention strategies [15].

The reviewed evidence also suggested that CHWs and the related cadres took part in a number of health promotion activities for malaria in various contexts [14, 15, 17–19]. Examples of such roles included counselling for malaria prevention, early treatment and improving health-seeking behaviour. They provided health education about malaria and related complications, prevention and treatment.

### Challenges of CHWs and related cadres in malaria interventions

Table 3 enumerates challenges and barriers CHWs and related cadres faced while implementing iCCM interventions. CHWs and related cadres faced health care financing challenges while implementing their roles in malaria interventions. This primarily included lack of sustainable sources of funds [20, 21]. As a result, CHWs and related cadres often suffered from poor or no remuneration [12, 22] and lack of incentives. Because the majority work on a voluntary basis, there has been no accountability when they are absent from the workplace [23].

**Table 3:** Challenges of CHWs, VMWs and lay personnel working on malaria

SN	Citation	Cadre	Challenges
1.	Rodriguez et al. [20]	Health surveillance assistants	Short training not in-keeping with medical regulation standards for prescription Lack of resources to lengthen training Poor supervision and overburden with patients Most are found in remote and hard-to-reach areas where frequent supervision is not routine Job description keeps changing with more introduction of community interventions Financial instability and poor sustainability because of donor dependence and other unreliable sources
2.	Chilundo et al. [21]	CHWs	Policy conflicts on prescription. Authority does not allow personnel with short-term training to prescribe Stock out of supplies especially anti-malarials Poor supervision especially in the hard to reach areas Funding instability. The programme is donor funded and subjected to delays in funding disbursement Lack of community involvement and ownership No evidence yet on impact and no evaluation strategy APEs are not paid
3.	Yansaneh et al. [33]	CHVs	CHVs are not remunerated and have to do other income generating activities Not available when needed as they are not paid for their service
4.	Nanyonjo et al. [30]	CHWs	Patients may not complete referrals
5.	Heidkamp et al. [26]	CHWs	Stock-out of essential supplies Poor supervision from higher cadres
6.	Druetz et al. [35]	CHWs	Community preference on qualified health workers CHWs not known to people Medicine stock-out Long distance to CHWs
7.	Banek et al. [13]	CMDs	Patients overload Lack of supervision Limited malaria knowledge Tensions with community members Lack of remuneration from the government
8.	Hamainza et al. [22]	CHWs	Lack of remuneration Negative attitudes to care given by CHWs Weak social responsibilities
9.	Abbey et al. [24]	CHWs	High attrition rate of CHWs especially in hard-to-reach areas
10.	Tine et al. [14]	CHWs	Medicine and RDT stock-out
11.	Ndiaye et al. [39]	CHWs	Medicine and supply RDT stock-out (ACT, RDT, gloves, case files, patients forms)
12.	Blanas et al. [28]	CHWs	ACT and other supplies stock-outs Expired medicines or unavailable in villages Scepticism from villages Transport problems, poor infrastructure and long distances for referrals
13.	Counihan et al. [25]	CHWs	RDT and other medical supply stock-outs after initial supplies finished Lack of supervision Sustainability
14.	Brenner et al. [23]	CHVs	Low turn-over of CHVs Low motivation Inconsistent supplies of medicine and supplies
15.	Gidebo et al. [66]	CHWs	Shortage of chloroquine, Patient pressure to take coartem
16.	Delacollette et al. [70]	CHWs	CHWs’ position remains ambiguous in the healthcare system. Non-comprehensive care may have negative effect on the sustainability of programme
17.	Ajayi et al. [77]	CHWs	*Challenges in their promotion/training activities* The community members were not in support of the project. Some community members felt trainers were wasting their time Trainers could not conduct training all the time because of their domestic needs

*CHWs* community health workers, *ASHA* accredited social health activist, *HCPs* home care providers, *CMDs* community medicine distributors, *VMWs* village malaria workers, *CORPs* community-owned resource persons, *CCDD* volunteer community-directed distributor, *VHVs* village health volunteers

Community health workers and related cadres have been facing similar health workforce challenges to other cadres working in malaria-related interventions. There has been a widespread lack of in-service training and other forms of continuous professional development [20]. Other related challenges include high turnover due to high attrition rates, especially for those working in hard-to-reach or remote areas [24], lack of incentives [23] and lack of motivation to continue with their work [12, 21].

Stewardship challenges also affected the role of CHWs and related cadres in malaria interventions. For example, in Malawi, abbreviated CHW training did not meet medical regulation standards for prescription resulting in CHWs not being allowed to prescribe anti-malarials [20]. Lack of supervision from qualified health workers and poor coordination from the existing health infrastructure affected implementation of CHWs’ role in iCCM [20, 21, 25, 26].

Lack of necessary medical supplies and medicine affected CHWs role in iCCM. Most studies mentioned stock-outs of ACT and other anti-malarials [21, 26, 27], test kits for malaria [13, 14, 25, 28] and gloves, among others [29].

Service delivery by CHWs working in malaria was impaired by a number of factors. First, CHWs and related cadres were not trusted to have adequate knowledge to care and treat malaria cases in some communities [21, 22, 27]. As a result, people who had symptoms of malaria still had to travel long distances to seek similar care in health facilities [27]. Second, distances from where they were stationed to households in need affected their service delivery [13], and the referral of their patients [30]. Third, lack of transport and poor roads caused delays in service delivery in some studies [13, 28].

Some of the iCCM and roles of CHWs and related cadres have not been evaluated [21]. This poses a challenge in scaling up this intervention to wider areas. Information and research are needed for understanding the challenges, lessons and areas for improvement when scaling up.

## Discussion

The current study is the first systematic review that summarizes evidence on the roles and challenges of CHWs and related cadres working on malaria interventions. In this review, CHWs and related cadres were already tasked with different roles in malaria interventions. They included prevention, malaria case management and health promotion related to malaria.

Community health workers and related cadres constitute the majority of potential health workforce for malaria and many other health-related interventions. Within the realm of malaria, understanding the breadth of their potential roles is an essential first step in order to best utilize the abundant pool of CHWs and related cadres. Their importance is augmented in the setting of human resource health crises, an overwhelming problem in most malaria-burdened countries due to their low-income country status [31]. The potential of utilizing CHWs and related cadres brings new hope in addressing both malaria and human resources for health challenges in such countries. This alternative resource can fill the gap if carefully tailored to suit the context [6] in order that efforts to control malaria and reduce morbidity and mortality can be achieved [7, 27].

Evidence presented shows a number of health system challenges [11] that CHWs and related cadres face. Such challenges have also been experienced in different settings with implementation of malaria interventions using other qualified cadres. The financial challenge is lack of stable funding to implement iCCM. In most settings of high malaria endemicity, malaria projects have been operating in donor-driven programmes that run vertically and were not integrated into the existing health system to ensure efficacy, timely delivery and to cut down bureaucracy. They have been expensive to run and lack sustainability beyond a project’s duration [32]. To ensure sustainability, CHWs and related cadres should be integrated into the health system infrastructure.

Short-term and focused training for CHWs and related cadres is a strength of iCCM. However, its cost effectiveness is a challenge in the implementation of malaria intervention, in particular, medical prescription and treatment [21]. It conflicts with other policies and regulations that require prescribers to have a minimum of training which is longer than that given to CHWs for iCCM [20, 32]. Short-term training reduces the community’s confidence in CHWs and related health cadres, which affects their utilization [22]. Tailor-made curricula for CHWs and related cadres should address conflicting policies and involve key stakeholders to ameliorate lack of confidence by the community.

Health workforce challenges are common among CHWs and related cadres. They include low or no remuneration, lack of recognition from some of the public health system, lack of incentives, and poor transport to remote areas. These are not uncommon causes of attrition, even among qualified medical and other health cadres. Addressing such challenges will help to deploy and retain CHWs and related cadres in hard-to-reach areas and solve the health workforce crisis in malaria-endemic areas.

Ensuring constant supply of anti-malarial and diagnostic tools, such as RDT and other supplies, is vital to implementation of iCCM. This review found that stock-outs were a common challenge. In some studies, the first consignment given after training of CHWs was never replaced when it ran out. To ensure reliable supply, health systems should incorporate CHWs and related cadres in malaria interventions as part of its strategy.

The evidence presented should be interpreted carefully owing to the following two limitations. First, meta-analysis could not be conducted on the retrieved evidence owing to differences in study designs and differences in outcome measures. However, the narrative synthesis was more suitable to this study to take advantage of different experiences and challenges encountered. Second, all lay health workers were included and combined together. Such health workers’ levels of knowledge, training duration, and context differed from one region to another. However, evidence generated has consistently shown similar roles and challenges of these cadres in malaria interventions.

## Conclusions

Community health workers and related cadres have been taking roles similar to those of more qualified health workers. They are important actors in malaria control and elimination but suffer from the health system challenges including financing, logistics, human resource management, and stewardship. To meet targets in sustainable development in health and to save countless lives and morbidity, CHWs and related cadres must be well resourced and sustained.
